# Concurrent acute myositis and Guillain-Barre syndrome in Cytomegalovirus infection – a rare case report

**DOI:** 10.1186/s12879-020-05506-5

**Published:** 2020-10-17

**Authors:** Varatharajan Sakthivadivel, Palanisamy Naveenraj, Arjun Kachhwaha, Deepak Kumar, Puneeth Babu Anne, Poonam Elhence, Mahadev Meena

**Affiliations:** 1 Department of Medicine, All India Institute of Medical Sciences Jodhpur, Jodhpur, Rajasthan 342005 India; 2 Department of Transfusion Medicine, All India Institute of Medical Sciences Jodhpur, Jodhpur, India; 3 Department of Pathology, All India Institute of Medical Sciences Jodhpur, Jodhpur, India

**Keywords:** Cytomegalovirus, Acute myositis, Guillain-Barre syndrome, Molecular mimicry

## Abstract

**Background:**

Cytomegalovirus (CMV) is a double stranded DNA virus and ubiquitous in nature. Association of Guillain-Barre syndrome (GBS) and CMV is well known but CMV acute myositis is a rare condition. Restriction of movements of limbs due to severe pain in myositis may obscure the diagnosis of GBS and this may easily miss.

**Case presentation:**

Here we describe a 29-year-old male presenting with pain and swelling of bilateral lower limbs which progressed rapidly with increasing serum creatine kinase levels with positive IgM CMV antibodies. In view of no improvement in clinical condition, patient was further evaluated and found to have concurrent GBS. He was treated with plasmapheresis and improved.

**Conclusion:**

Cytomegalovirus infection presenting as acute myositis is a uncommon and further association with GBS is a rare occurrence.

## Background

Cytomegalovirus (CMV) associated neurological manifestations range from encephalitis, meningitis, myelitis to polyradiculopathy and rarely multifocal neuropathy [1]. Guillain-Barre syndrome (GBS) is an autoimmune polyradiculoneuropathy and its association with recent infections like Campylobacter jejuni, CMV, Epstein-Barr virus (EBV) and Mycoplasma pneumonia is well established. GBS is the most common acute neuropathy in adults with incidence of 1.3 per 100,000 [2]. The pathogenesis of GBS involves autoimmune response to preceding infection. This autoimmunity initiates multifocal inflammation in myelin sheaths of the spinal roots and peripheral nerves [3].

Acute myositis has a wide range of aetiology, in which viral myositis is most common. CMV as a cause of acute myositis is rare and commonly reported with immunosuppressive conditions like HIV and solid organ transplant [4]. Muscles are affected either directly by invasion of virus or may be damaged by inflammatory cytokines and autoantibodies triggered by the virus. Acute myositis, caused by CMV infection, leading to severe rhabdomyolysis even in immunocompetent individuals has been reported [5].

Occurrence of concurrent GBS and acute myositis have rarely been reported in the literature with dengue fever and mycoplasma pneumoniae infection [3, 6] but not with CMV infection. Here we report of concurrent development of GBS and acute severe myositis with rhabdomyolysis in a young male presenting with acute paraparesis and muscular pain.

## Case presentation

**Fig. 1:**
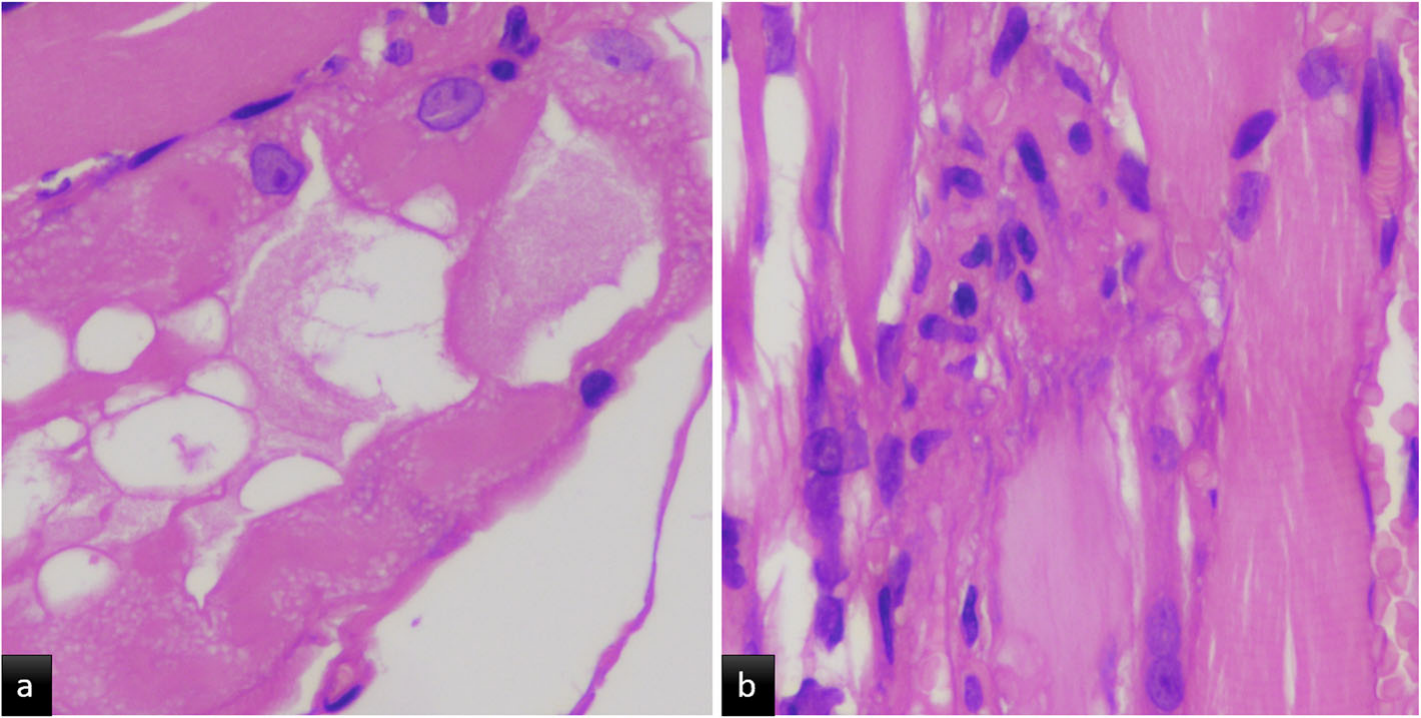
**a** Cross section of skeletal muscle fiber, Moth eaten and vacuolated appearance, 40x, H&E stain (Hematoxylin and Eosin). **b** Occasional myophagocytosis with histiocytic cells, 40x, H&E stain (Hematoxylin and Eosin)

A 29-year-old previously healthy male, presented with complaints of mild fever for 5 days, which subsided with paracetamol. After 3 days of asymptomatic period, he started having acute onset severe dull aching diffuse pain in both lower limbs with swelling and difficulty in walking. He also reported oral ulceration and difficulty in swallowing. He was brought to the emergency room on wheelchair and was unable to stand-up on his own. On examination, his vitals were stable. He had oral thrush. Lower limbs were edematous and tender. Neurological examination revealed decreased power grade (2/5) and absent reflexes in both lower limbs. Superficial reflexes were absent including plantar response. His investigations revealed normal complete blood count (Hb – 17.8 g/dL, Total Leucocytes count – 6600/μL, platelet count – 357,000/ μL), and deranged liver function (ALT- 220 IU/L and AST = 549 IU/L). His renal functions were within normal limits. There was marked increased in creatine kinase (11,960 U/L initially and 24,240 U/L after 1 day) and lactate dehydrogenase levels (1747 U/L). A presumptive diagnosis of acute myositis was made with movement restriction due to severe pain. He denied any history of trauma, drug abuse or toxin exposure. A viral panel for CMV, EBV, HCV, HIV, HbsAg and Dengue virus were sent along with blood and urine cultures. The results showed presence of CMV IgM antibodies by ELISA only. Leptospirosis and scrub typhus were also ruled out with relevant test. The diagnosis of acute CMV myositis was made and the patient was treated with oral valganciclovir (900 mg twice daily). The oral prednisolone 60 mg/day which was started on day one with suspicion of acute idiopathic myositis stopped after positive CMV IgM antibody. After 3 days of treatment there was improvement in pain and tenderness dramatically but no improvement in power of lower limbs. Nerve conduction velocity (NCV), electromyography (EMG) and lumbar puncture were done to rule out GBS. NCV was suggestive of demyelinating of sensory and motor nerves and also sensory axonal pattern of involvement with albumino-cytological dissociation in cerebrospinal fluid (protein – 80 mg/dl, WBC - 4 cells/mm^3^). EMG showed active acute denervation potentials with no motor unit potentials in quadriceps and, neurogenic changes with no definitive myopathic potentials in biceps. Muscle biopsy from left vastus lateralis was suggestive of focal myonecrosis with occasional regenerative fibres and myophagocytosis (Fig. 1a &b). Hence, a final diagnosis of acute myositis with GBS was made. Patient was treated with five sessions of alternate day plasmapheresis and showed dramatic improvement in power of lower limbs (Grade 4/5). Creatine kinase had decreased to 1215 U/L. Blood samples for CMV PCR came negative but convalescent phase sampling was positive for IgG CMV antibodies in high titre by ELISA. In view of oral candidiasis human immunodeficiency virus test were done, which was negative. However, his CD4 count was only 127 cell/mm^3^ raising possibility of associated idiopathic CD4 lymphocytopenia. We are planning to rule out this by repeating CD4 counts after 3 months. Patient was discharged with advice to continue follow-up in medicine and physical medicine and rehabilitation department.

## Discussion and conclusion

Cytomegalovirus is a double stranded DNA virus belonging to the Herpesviridae family (HHV-5). Clinically significant CMV disease usually occurs in immunocompromised patients in whom it can affect almost every organ. Most common manifestation of CMV in immunocompetent individual is mononucleosis syndrome [7].

Acute viral myositis is an uncommon manifestation of CMV and direct infection of muscles and autoimmunity are likely pathogenesis. In literature, less than 10 cases of CMV myositis have been reported and the maximum number of cases were from younger than 40 years [5, 8–12]. Muscle pain associated with high creatine kinase and muscle biopsy are diagnostic. Laboratory diagnosis was confirmed by seroconversion from IgM to IgG but CMV PCR was negative in maximum reported cases. Our case also had muscle pain, high creatine kinase, seroconversion from IgM to IgG and muscle biopsy favours myonecrosis which confirms the diagnosis of acute myositis. We had ruled out toxin, trauma, drugs, autoimmune disorder and other viral causes. Rhabdomyolysis is a dreaded complication of acute myositis which is also true for CMV myositis. Rhabdomyolysis is a skeletal muscle injury, characterized by release of intracellular muscle cell contents that includes electrolytes, myoglobin, creatinine kinase, LDH and aminotransferases in the blood [13]. Our patients had severe pain and swelling in lower limbs with high creatine kinase and high LDH level indicating extensive muscle damage. Urine myoglobin can be used for diagnosis of rhabdomyolysis but detected in only 19%, so the absence of urine myoglobin doesn’t exclude rhabdomyolysis [14]. However, muscle biopsy confirmed necrosis of muscle fibres with myophagocytosis. Similar cases of acute myositis, with muscle biopsy showing necrotic fibres with myophagocytosis have been reported and the patients recovered well over a period of 2 weeks with intravenous corticosteroids and supportive treatment [15].

Guillain-Barré syndrome is a rapidly progressive polyradiculoneuropathy with a monophasic course. Most of the patients have symptoms of preceding infection within 4 weeks of initiation of weakness [16]. Our patient had fever 3 days prior to onset of weakness. Most common associated infection with GBS is Campylobacter jejuni followed by CMV. Molecular mimicry is the suggested pathogenesis, in which cross reacting antibodies to non-self-antigen, bind to the antigens exposed on Schwann cells or axons of the nerve. Anti GM2 antibodies are often associated with CMV induced GBS [17]. Antibodies once bound cause complement activation which lead to formation of Membrane Attack complex (MAC). Once there is damage to myelin sheath of axons, macrophage invasion occurs to clear the debris [18]. Duration for regeneration of neurons and return to normal function depends on the extent and severity of damage. Patients with CMV related GBS are generally younger and have severe disease than idiopathic forms of the syndrome [19]. Our patient was also young and had severe disease. The specific clinical characteristics in CMV associated GBS include sensory involvement, respiratory muscle involvement and cranial nerve palsies. The long-term neurological sequelae are also less severe than other causes of GBS [20]. In our case, the patient had both motor and sensory nerve involvement. Nerve conduction study shows non-excitable motor and sensory nerves with axonal pattern of involvement, suggestive of Acute motor sensory axonal neuropathy (AMSAN) variant of GBS. Proven modalities for treatment are intravenous immunoglobulins and plasma exchange and best results can be seen if initiated within 2 and 4 weeks of onset of weakness [16].

Concurrent GBS and myositis have been reported in mycoplasma pneumoniae and dengue virus infection in literature [3, 6]. GBS associated with fatal rhabdomyolysis has been reported but not with CMV [13]. Possible cause of association in both case reports were also autoimmunity and PCR for microorganism was negative [3, 6]. Concurrent GBS with myositis is not reported in CMV and autoimmunity and molecular mimicry could be the possible pathogenesis. Direct invasion of muscle and nerves by virus could be another possible pathogenesis. However preceding symptomatic infection and negative CMV PCR with positive IgM favours the autoimmune pathogenesis. A dramatic improvement after plasmapheresis also pointed towards autoimmunity.

Cytomegalovirus infection usually manifests clinically only in immunocompromised persons like HIV patients, post-transplant patients and those on long term immunosuppression. Our patient had oral candidiasis which suggests immunodeficient state. As the common immunodeficiency states were ruled out, idiopathic CD4 T-cell deficiency may be considered as a cause. Absolute CD4 count is yet to be repeated after 3 months for confirmation of the same.

In conclusion**,** Cytomegalovirus infection presenting as acute myositis is a uncommon and further association with GBS is a rare occurrence. This case highlights the importance of clinical vigilance and judgement to consider it as a differential diagnosis which may significantly alter the treatment approach. The molecular mimicry is thought to be probable pathogenesis of this rare association. This infection though common in immunocompromised individuals, is a possibility in immunocompetent and a high degree of suspicion may help in prompt diagnosis and treatment.

## Data Availability

The datasets used and/or analysed during the current study available from the corresponding author on reasonable request.

## Abbreviations

CMV: Cytomegalovirus
GBS: Guillain-Barre syndrome
EBV: Epstein-Barr virus
HIV/AIDS: Human Immunodeficiency Virus/Acquired Immunodeficiency Syndrome
ALT: Alanine transaminase
AST: Aspartate aminotransferase
HCV: Hepatitis C Virus
HbsAg: Hepatitis B Surface Antigen
NCV: Nerve conduction velocity
EMG: Electromyography
ELISA: Enzyme-linked Immune Sorbent Assay
LDH: Lactate dehydrogenase
MAC: Membrane Attack complex
AMSAN: Acute motor sensory axonal neuropathy
PCR: Polymerase Chain Reaction

## References

[1] Anders HJ, Goebel FD (1999). Neurological manifestations of cytomegalovirus infection in the acquired immunodeficiency syndrome. Int J STD AIDS.

[2] Merzkani M, Israel E, Sachdeva M (2017). Primary Cytomegalovirus infection causing Guillain-Barré syndrome in a living renal allograft recipient. Case Rep Transplant.

[3] Topcu Y, Bayram E, Karaoglu P, Yis U, Guleryuz H, Kurul SH (2013). Coexistence of myositis, transverse myelitis, and Guillain Barré syndrome following mycoplasma pneumoniae infection in an adolescent. J Pediatr Neurosci.

[4] Crum-Cianflone NF (2008). Bacterial, fungal, parasitic, and viral myositis. Clin Microbiol Rev.

[5] Gindre H, Féasson L, Auboyer C, Cathébras P (2013). Severe rhabdomyolysis associated with a primary cytomegalovirus infection in an immunocompetent patient. BMJ Case Rep.

[6] Gulia M, Dalal P, Gupta M, Kaur D (2020). Concurrent Guillain-Barré syndrome and myositis complicating dengue fever. BMJ Case Rep.

[7] Kano Y, Shiohara T (2000). Current understanding of cytomegalovirus infection in immunocompetent individuals. J Dermatol Sci.

[8] Sato K, Yoneda M, Hayashi K (2006). A steroid-responsive case of severe rhabdomyolysis associated with cytomegalovirus infection. Rinsho Shinkeigaku.

[9] Maeda M, Maeda A, Wakiguchi H (2000). Polymyositis associated with primary cytomegalovirus infection. Scand J Infect Dis.

[10] Yasumoto N, Hara M, Kitamoto Y (1992). Cytomegalovirus infection associated with acute pancreatitis, rhabdomyolysis and renal failure. Intern Med.

[11] Hugues GS, Hunt R (1984). Cytomegalovirus infection with rhabdomyolysis and myoglobinuria. Ann Intern Med.

[12] Hirohama D, Shimizu T, Hashimura K (2008). Reversible respiratory failure due to rhabdomyolysis associated with cytomegalovirus infection. Intern Med.

[13] Saxena A, Singh V, Verma N (2014). Guillain-Barre syndrome complicated by acute fatal rhabdomyolysis. Indian J Crit Care Med.

[14] Melli G, Chaudhry V, Cornblath DR (2005). Rhabdomyolysis: an evaluation of 475 hospitalized patients. Medicine (Baltimore).

[15] Rengaraj R, Arulmozhi T, Dhanaraj M (2006). Acute myositis. J Assoc Physicians India.

[16] Willison HJ, Jacobs BC, van Doorn PA (2016). Guillain-Barré syndrome. Lancet..

[17] Khalili-Shirazi A, Gregson N, Gray I, Rees J, Winer J, Hughes R (1999). Antiganglioside antibodies in Guillain-Barré syndrome after a recent cytomegalovirus infection. J Neurol Neurosurg Psychiatry.

[18] Hughes RA, Cornblath DR (2005). Guillain-Barré syndrome. Lancet..

[19] Orlikowski D, Porcher R, Sivadon-Tardy V, Quincampoix JC, Raphaël JC, Durand MC (2011). Guillain-Barré syndrome following primary cytomegalovirus infection: a prospective cohort study. Clin Infect Dis.

[20] Visser LH, van der Meché FG, Meulstee J, Rothbarth PP, Jacobs BC, Schmitz PI (1996). Cytomegalovirus infection and Guillain-Barré syndrome: the clinical, electrophysiologic, and prognostic features (Dutch Guillain-Barré study group). Neurology..

